# Extending the authority for sickness certification beyond the medical profession: the importance of ‘boundary work’

**DOI:** 10.1186/1471-2296-15-100

**Published:** 2014-05-17

**Authors:** Victoria K Welsh, Tom Sanders, Jane C Richardson, Gwenllian Wynne-Jones, Clare Jinks, Christian D Mallen

**Affiliations:** 1 Arthritis Research UK Primary Care Centre, Primary Care Sciences, Keele University, Keele, Staffordshire ST5 5BG, UK

**Keywords:** Professional boundaries, Sick certification, Qualitative methods, Sociology of professions, Primary care

## Abstract

**Background:**

The study aimed to explore the views of general practitioners (GPs), nurses and physiotherapists towards extending the role of sickness certification beyond the medical profession in primary care.

**Methods:**

Fifteen GPs, seven nurses and six physiotherapists were selected to achieve varied respondent characteristics including sex, geographical location, service duration and post-graduate specialist training. Constant-comparative qualitative analysis of data from 28 semi-structured telephone interviews was undertaken.

**Results:**

The majority of respondents supported the extended role concept; however members of each professional group also rejected the notion. Respondents employed four different legitimacy claims to justify their views and define their occupational boundaries in relation to sickness certification practice. Condition-specific legitimacy, the ability to adopt a holistic approach to sickness certification, system efficiency and control-related arguments were used to different degrees by each occupation. Practical suggestions for the extension of the sickness certification role beyond the medical profession are underpinned by the sociological theory of professional identity.

**Conclusions:**

Extending the authority to certify sickness absence beyond the medical profession is not simply a matter of addressing practical and organisational obstacles. There is also a need to consider the impact on, and preferences of, the specific occupations and their respective boundary claims. This paper explores the implications of extending the sick certification role beyond general practice. We conclude that the main policy challenge of such a move is to a) persuade GPs to relinquish this role (or to share it with other professions), and b) to understand the ‘boundary work’ involved.

## Background

In the UK, demand on primary health care continues to rise as the population ages, health reforms focus on shifting secondary care services into the community and the service delivery targets continue to be developed, for example the linking of physician pay to outcomes as with the Quality Outcomes Framework [1]. Role extension, defined as the ‘substitution of doctors’ traditional role’ can be a useful means of achieving increasing healthcare system efficiency [2]. In the current setting, this is particularly relevant to primary health care teams, who have evolved to include extended roles for non-medical health professionals including nurses and physiotherapists. Indeed, primary care nurses are increasingly becoming the first point of contact for healthcare and are managing chronic disease [3] and physiotherapists often manage patients with minimal input from the general practitioner [4].

Role extension in primary care has generally met positive reviews with advanced roles of primary care nurses deemed successful [5]. Physiotherapist role extension to act as first point-of-contact practitioners has received high levels of support from GPs and physiotherapists [6]. However, concerns over the negative impact of role extension in primary care have been raised. For example, previous research highlights that cost savings achieved through the substitution of doctors for primary care nurses may be offset by the lower productivity of nurses and potential increase in doctor-workload due to nurses meeting previously unmet needs or generating demand for care where previously none existed [6].

Boundaries between professions are fundamental to professional identity and as such, occupations often undertake ‘boundary work’ to maintain such identity. By ‘boundary work’ we refer here to the process by which professions attempt to maintain ownership over a sphere of work. In previous studies such practices included the process by which occupations made claims to specialist knowledge or through direct negotiation between occupations to demarcate work boundaries [7-9]. In the context of changing healthcare policies and organisational structures, boundary work is particularly important to maintain control over a sphere of work. Although there is much interest in evaluating extended roles in primary care, there is a paucity of literature exploring the impact of boundary work on role extension in primary care.

The research presented here sought to explore the views of GPs, practice nurses and physiotherapists towards the extension of sickness certification beyond the medical profession, to identify areas of consensus and disagreement. The notion of ‘boundary work’, a key component of which is the idea of ‘legitimacy claims’, was used as a supporting theory to explain attitudes towards the role extension concept by three groups of health care professionals.

### Professional boundaries

The literature on professional boundaries shows that jurisdictions must be legitimated in the context of where professionals carry out their work and interact with other professions [10,11]. The manner of these legitimization practices has taken many forms, indicating the often subtle ways that occupational groups attempt to assert authority over the content and scope of their work. For instance, groups may attempt to protect their claims to a specific jurisdiction through the delegation of ‘unwanted’ tasks to others; thus general medical practitioners (GPs) may pass on routine work to nurses whilst seeking to retain their overarching status as ‘expert’ [12,13]. Abbott [10] argued that by the late 20^th^ Century many professions had come to rely heavily on science as a means of legitimation. In this context, ‘science’ includes both its narrow definition and a broader understanding as rationality and efficiency, a point to which we return below. Several studies have highlighted how distinctions are made when practitioners appeal to the scientific basis of their work [14,15]. Science narrowly conceived is, however, not static. Increasing sub-specialisation in medicine is perhaps one manifestation of this [16]. In any event, such practices always have ‘discursive’ characteristics.

For all of these reasons, we might expect such discourses in the workplace to be dynamic and opportunistic. One possibility is that such discourses exhibit a moral content, the bases of which might range from judgments about different patient groups as ‘good or rubbish’ [17] to ‘atrocity stories’ in which health visitors distinguished themselves from doctors with reference to the negative attitudes of the latter [18]. A second possibility is that the notion of ‘science’ might be reconceptualised or reused in various ways. For instance, the notion of ‘clinical isomorphism’ [19] has been used to signify the readiness of one health profession to adopt the scientific norms of another. Thus, rather than emphasising the relative merits afforded by alternative therapy within the hospital setting (such as its holistic character) practitioners in Mizrachi et al’s [11] study sought acceptance from medicine by indicating a need for scientific research to validate the effects of their therapies, and often referred to patients as ‘cases’, indicating that they were adopting a medical discourse. The rhetorical reduction of the patient to a medical case was therefore indicative of their desire to emulate. A rather different example is the broadening of the notion of ‘science’ to encompass wider rationalities such as the effectiveness and efficiency of treatments, or adherence to research-based clinical protocols [10,20]. Of course, it is not necessarily the case that professions are consistent in their legitimation discourses; Foley and Fairclough [21] found that midwives used discourses of both ‘medicine’ and ‘collaboration’, which they deployed in different ways depending on the context of their work. They reported that use of the language of medicine by midwives was an attempt to establish themselves as equal to doctors, because they too used ‘science’ in their work. However, at other times they placed themselves in a cooperative relationship with physicians as a means of validating their location in the professional status hierarchy.

For all of the above reasons professional work boundaries need to be considered in any analysis of occupational behaviour change, to acknowledge the wider context of the NHS multidisciplinary workforce. The extension of sickness certification to professional groups other than medicine is likely to result in considerable inter-professional boundary negotiation.

### Sickness certification in the UK context

In 2011, 131 million days were lost due to sickness absences in the UK [22], costing the UK economy £17 billion [23]. Furthermore, 2.07 million adults of working age were out of work due to long-term sickness absence in 2010 (ONS [22]). For those unable to work due to ill-health, sickness certificates provide supporting evidence for health-related benefits claims [24]. In the UK, only medical doctors are legally able to certify sickness absence [25] despite evolving extended roles within the primary health care team. GPs are contractually obliged to certify short- and medium-term sickness absence [26]. Estimates suggest GPs spend six consultations every half-day session discussing work and health and a full-time GP expects to sign approximately ten sickness certificates every week [27]. In 2001, the Government estimated that extending the authority to certify sickness absence to primary care nurse practitioners would save 2.4 million GP appointments and 51,000 hours of GP time per year. As demands are increasingly placed upon GPs, role extension in sickness certification is an important proposition to consider, particularly as GPs hold mixed views towards their sickness certification role. Some GPs value their participation and feel they are best placed to fulfil this role, others prefer to have the role removed [28]. Recently, a majority of surveyed GPs thought primary care nurses and physiotherapists should have the authority to certify at least some sickness absence [27].

Evidence demonstrates that work is generally good for health, yet the predominant national philosophy that illness is incompatible with work remains [29,30]. In light of this evidence, the Government introduced a strategy on health, work and wellbeing to encourage and assist individuals with ill-health to return to work [29,30]. Part of this strategy, the ‘Fit for Work Service’, emphasises a multidisciplinary approach in encouraging an early return to work which includes increasing responsibilities placed upon nurses and physiotherapists. Government policy on managing health and work is becoming more proactive, as opposed to passive in providing disability benefits. There has been the introduction of the Fit for Work Service [24] and a new White Paper Fitness for Work: The Government response to “health at work” has set out the Government’s policy in relation to the development of a health and work advisory service providing access to state funded occupational health, improving sickness absence management in the workplace alongside support for healthcare professionals and a reform of the benefits system [31]. However, a shift towards proactive health and work management in the UK means that diverse skills are required to help people stay in work and manage their work related difficulties. One potential barrier to this could be the possible reluctance of GPs to relinquish their responsibilities for managing work and health to other occupational groups (eg. primary care nurses) or to share this role.

In light of re-focused UK Government priorities towards work and health, the current role extension in primary care and renewed support for role extension in sickness certification to nurses and physiotherapists, this paper seeks to further explore views towards primary care sickness certification role extension, and the potential practical benefits and barriers such a move would involve. We report the views of practice nurses, physiotherapists and GPs to establish whether there is support for role extension, and if so, the key challenges to its introduction. We focus specifically on the relevance of occupational boundaries in role extension policy introduced in the NHS.

### Sickness certification: the international context

Research on the role of health professionals and their attitudes towards sickness certification is scarce, and international comparisons indicate wide variations in beliefs and corresponding behaviours among patients and primary care practitioners [32-38]. Research from Scandinavia has made significant progress in understanding GPs’ attitudes towards sickness certification and work absence. One study found that the strongest indicator of sickness certification is the extent of concordance between patients’ and GPs’ evaluations of reduced work capacity [39], whilst a diagnosis of musculoskeletal disease or mental illness increased the likelihood of work absence; perhaps reflecting societal pressure and expectation to exempt people with certain health conditions from participation in work [40]. Other studies from Scandinavia highlight the difficult challenge of fitness for work assessments in the presence of clinical uncertainty about the patients’ presenting complaint, particularly in the absence of objective signs [41]. In such circumstances, GPs may be inclined to accept the patient’s complaint and issue a sick certificate [39]. Research from Scandinavia and the UK also shows that GPs consider work related issues to be less relevant to their primary role and may be ill equipped to assess people’s capacity to work [42,43]. In a recent survey, almost two thirds of employers claimed that occupational health specialists, not GPs, were best placed to assess people’s fitness to work [44]. It is clear from the international literature that significant variation exists in GPs’ assessments of work capacity and decisions to issue a sick certificate. Given the cost implications for global economies, an improved understanding of how the delivery of sickness certification could be improved is needed, and this includes more in-depth research on the possibility of extending authority to other healthcare practitioners [45-48].

More recently, research in the UK and abroad has begun to examine the impact of illness on work absence, placing greater emphasis on the broader role of employers and organisational policies in facilitating people’s return to work [49-51].

## Methods

A random sample of 125 GPs were selected from a list of 397 GPs who consented to receiving further study invitations as part of a previous research study [27]. Purposive sampling was subsequently employed to select 15 GPs from the 26 who consented to participate. Participants were selected on the basis of practice location, practice list size, service duration, postgraduate occupational health qualifications, contract basis (partner, salaried, locum, full-time, or part-time) and sex. Nurses and physiotherapists were recruited through snowball sampling. Nurses were recruited through their GP colleagues to explore the influence of working relationships and common working environments upon views. Five nurses were matched to their GP colleagues and two were unmatched. The reason for this was that we could not recruit all nurses in the same practices as their GP colleagues, though five matched pairs offered a useful insight into how the views towards role extension among GPs and nurses working together compared. As with any ‘snowball’ sampling approach, it is possible that participants sharing a common working space may hold similar or ‘compatible’ attitudes towards a particular working pattern or behaviour. At the same time such views may help to better understand the underlying reasons and whether and to what extent these are influenced by local organisational factors, or whether attitudes towards role extension are largely formed on the basis of professional differences or training. Six physiotherapists were randomly recruited through local research networks. The topic guide used broad prompts to explore views towards GP roles, views on role extension and practical requirements of role extension. One researcher (VKW) undertook semi-structured telephone interviews lasting 30–60 minutes. Written informed consent was obtained for study participation, interview recording and quotation use. Interviews were transcribed verbatim.

A total of 28 respondents were interviewed. Nine GPs were male, the median time in practice was 21 years (range: 5–32 years), ten worked full-time, eleven were practice partners (three were salaried GPs, one worked as a locum) and three had advanced occupational health training. All the nurses were female, the median time in practice was 14 years (range: 2-33 years) and four had advanced training, although not in occupational health. The GPs and nurses worked in a range of large, medium and small practices in cities and towns. Four of the physiotherapists interviewed were male, which is not representative of the average proportion of male physiotherapists in UK clinical practice. Although male physiotherapists may differ in their views to some extent, it is unlikely that their perceptions about role extension in relation to sickness certification would dramatically deviate from female physiotherapists. The median time in practice was seven years (range: 1-40 years), five worked full-time and all worked in multiple locations. Two physiotherapists possessed advanced training in occupational health and four had advanced training in musculoskeletal health. Fifteen respondents were interviewed at work and 13 were interviewed at home. New themes ceased to emerge after 13 GP and six nurse interviews, but continued to emerge during the final physiotherapist interview. Respondent characteristics such as age, sex and training in occupational health did not appear to influence opinions towards a particular viewpoint; either in favour or against role extension. Despite preference towards support for role extension, notable exceptions from each professional group existed. Support for role extension did not always mean a rejection of the GP sickness certification role. As with any qualitative study, the ultimate aim is to explore participants’ views in depth rather than to achieve generalizable findings. We over recruited GPs into the interview study because they are primarily responsible for issuing sickness certificates in the UK, and therefore we sought to utilise their views as a starting point for our analysis, and using the nurse and physiotherapist interviews to compare and contrast the views of GPs. Moreover, limited in-depth qualitative evidence exists of GPs’ perceptions of sickness certification and attitudes to service re-design such as role extension. Although a smaller number of nurses and physiotherapists were recruited into the study, the data had reached ‘saturation’ point in relation to the *role extension concept* (if not in relation to other less directly relevant issues) and thus we decided that further interviews were not needed. A topic guide was used (the same for each participant group) which included questions on how participants approached sickness certification decisions during clinical practice, their views towards extending or sharing responsibility for such decisions, and in which circumstances role extension would be appropriate. The topic guide was amended to some degree during fieldwork as new insights and themes emerged from the interviews. These new themes were subsequently included in the topic guide and explored in the remaining interviews. One example of a new theme to emerge from the initial interviews was the idea of ‘boundary work’ and participants’ claims to the jurisdiction of ‘sickness certification’ practice. The decision to end fieldwork was based on ‘thematic saturation’, where new insights in relation to the research question were no longer emerging from the interviews.

The main author’s background as a general practitioner with a special interest in social science influenced the theoretical focus taken to the research question and analysis of the data. However, the concept of ‘boundary work’ emerged from the analysis and we reviewed the literature on professional boundaries during the course of the fieldwork in order to interpret the findings. The initial aims of the research were not to investigate ‘boundary work’ but to explore different views towards role extension in relation to the sickness certification role by three groups of healthcare professionals.

Data analysis was continuous and iterative throughout data collection to enable exploration of emerging themes. Thematic analysis was undertaken using constant comparative methodology, facilitated by NVivo9 to code and organise the data. The first transcript was independently coded by two researchers (VKW, JR). The initial codes were discussed and revised so that agreement about their appropriateness was reached by the analysis team. These codes were applied to several transcripts, followed by discussion and comparison. Any differences in coding were discussed until a consensus was reached. The emerging coding frame was applied to the remaining transcripts by a single researcher (VKW). Themes were compared across participants and within individual accounts. The four key themes arose directly from the data analysis. The research team reached consensus about the interpretation of these themes and how they might best be ‘labelled’ and defined. The concept of ‘legitimacy claims’ which is a key component of ‘boundary work’ carried out by healthcare professionals seemed to provide an appropriate theoretical ‘lens’ for interpreting our findings. The data cannot strictly speaking be viewed as a direct reflection of clinical practice because we have relied entirely on the perceptions of three occupations rather than observed behaviour. Each occupational group may therefore have their own motives and agendas to support their particular views about sickness certification, and may not necessarily represent ‘how things are done’ in daily clinical practice. However, the data lend itself to an exploration of professionals’ diverse agendas and motives towards extending the sick certification role, and which revealed a number of barriers and possibilities for introducing service redesign in primary care. Ethics approval was obtained from a local NHS ethics committee.

## Results

**Figure 1:**
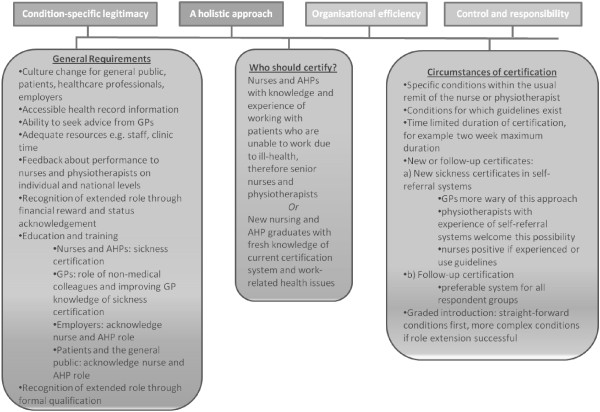
Suggested requirements for extended role sickness certification implementation and their underlying professional legitimacy claims.

Respondents employed four key arguments, or ‘professional legitimacy claims’, to maintain professional boundaries and ultimately, to maintain professional identity. Practical suggestions for implementation (Figure 1) are explained by professional legitimacy claims. A unique numerical identifier is included at the end of each quotation to identify interview participants.

### Condition-specific legitimacy

Respondents based their claims on perceptions of their own and other professions’ specialist expertise to justify their part in the certification process and the role extension concept. Narratives of each professional group were based around the distinction between *“straight-forward”* and *“woolly”* medical problems.

GPs highlighted their skill in managing complex multimorbidity, indicating that sickness certification by nurses or physiotherapists for such cases would be less acceptable whilst nurses reinforced GP views of nurses certifying straight-forward cases.

*“…if you’ve got more complicated cases, I just don’t think that would be suitable for a nurse… it’s very complicated, it’s not easy to get the patient back to work. You have to be careful that you’re happy that the patient is fit and sometimes there are lots of other psychological reasons why they don’t want to return to work.”* GP 304

*“I suppose it depends on what the condition was. If someone came in with chronic back pain, obviously we couldn't assess that person. But if it was for…fairly self-limiting illnesses…one of our patients had a really nasty insect bite, worked in a lab. We'd been seeing him regularly. So in that situation I guess we could have done a sick note…”* Nurse 321

Physiotherapists, however, employed their specialist knowledge to define their role and alluded to GPs’ knowledge gap to legitimise role extension claims:

*“…because physiotherapists are at the heart of the rehabilitation process, the person needs to have time off work to help their recovery, we could identify those patients quite well… I think your average GP can sometimes struggle with just assessing a hip, if it needs to be replaced or not and to then identify if that person’s got any emotional overlay on top of that hip pain…I just don’t think it’s in their remit….”* Physiotherapist 11

A strong sense of certification within usual clinical remit emerged, reinforced by matched pairs presenting similar arguments of condition-specific legitimacy. The Table 1 below presents situations that respondents deemed “*appropriate”* for extended role certification.

**Table 1:** Medical problems deemed ‘appropriate’ for extended role sickness certification by GPs, nurses and physiotherapists

Primary care nurses	Wound care including chronic ulceration, animal bites
	Self-limiting medical conditions including chest infections, urinary tract infections,
	Chronic disease management including diabetes, chronic obstructive pulmonary disease, cardiovascular disease
Physiotherapists	Musculoskeletal conditions including low back pain, tennis elbow, shoulder pain, knee pain, acute injuries, chronic pain

### A holistic approach

All professions recognised the need to adopt a holistic approach towards sickness certification. Each group appeared to equate *“the full picture”* with the ability to practice holistically. Information access seemed a central requirement of holistic practice.

GPs legitimised their certification role and defined their occupational boundaries through highlighting their monopoly over the holistic approach:

*“Of course you have the advantage that you usually know the patient very well so you know a lot more of the background…that does help…we see everything that goes on with the patient and that puts us in a unique position, we get a complete overview of the patient. Whereas, a physiotherapist is, understandably, more specialist in that area so they’re not going to see the whole patient the same way that we are.”* GP304

Additionally, GPs referred to their gatekeeper role to patient information through raising concerns over the appropriateness of fit note completion without access to full records and the ethical challenges of information sharing:

*“When you get referred to a physio, generally they're focusing on one element. It may be straightforward if they're seeing someone rehabilitating from a total knee replacement or something but it's not always that straightforward and they're not likely to know the background issues at all…they'll have the sketchiest of referral information but they may never have been referred from primary care. So they're not forced to know anything about the background. And our notes might be writing ‘patient clearly fit enough to work. Needs to return. Told I will not issue any sick notes anymore’ and they won't have any access to that record.”* GP760b

*“…it’s only ever been GPs who are at the centre of all the networks of information about a patient and there would be patient confidentiality problems if you were relaying it. It would be unnecessary to relay it as you’ve got it all.”* GP145

Physiotherapists similarly acknowledged their inability to access primary care records due to geographical location and the mobile nature of their work. They attributed the lack of information access as a limitation in holistic practice and thus ability to make informed decisions over sickness certification. One physiotherapist described this obstacle to holistic practice as ‘insurmountable’. Thus, physiotherapists equated access to patient information held by GPs with holistic care.

Nurses and physiotherapists legitimised their role extension by highlighting their ability to spend more time with patients than GPs, thus achieving a *“more in depth”* approach to *“get to the bottom of what is going on”*. In contrast to his colleagues, one GP stated how reduced consultation times and increasing target-driven medicine precluded his ability to practice holistically and therefore undertake sickness certification.

### System efficiency

All professional groups recognised the potential of role extension to increase system efficiency including GP workload reduction, improved healthcare access for sickness certification and removal of work duplication.

Despite their contrasting views towards role extension, GPs employed this claim to support *“streamlining the fairly obvious”* cases to others to manage to save GPs time and enable them to focus on more *“urgent”* patient problems. Examples of scenarios in which role extension would improve organisational efficiency included patients requiring post-operative monitoring by nurses (for example, wound dressings or stitch excision); patients receiving a diagnosis and management plan from other frontline healthcare providers (for example, the emergency department and walk-in-centres) who only have to attend the GP specifically for a sickness certificate; patients receiving a diagnosis, management plan and follow-up from a nurse practitioner but have to see the GP for a sickness certificate (for example, chronic disease monitoring); minor illnesses and injuries (for example, gastroenteritis and tonsillitis) and patients undergoing treatment with the physiotherapist who only needed to see the GP specifically for a sickness certificate. Every matched pair gave similar narratives about improving efficiency, indicating that shared values may arise from a shared organisational culture.

GPs seemed to use system efficiency arguments as an indirect means of exercising influence, defining other occupations’ ‘acceptable’ roles. GPs may support role extension if it saves them time, not because physiotherapists or nurses are the better professions to do it.

Physiotherapists supported their legitimacy claims to role extension through suggesting it *“would save an extra trip to the doctors”.* Nurses employed accessibility to healthcare to support their role extension claims by highlighting the relative ease of obtaining nurse-appointments.

All nurse and five physiotherapist respondents viewed sickness certification as a natural role progression:

*“…for me, it would seem like a natural progression of my role…I’ve done everything else: they’ve come in, I’ve assessed them, we’ve discussed what their treatment options could be, we’ve decided on a plan, I’ve prescribed and printed out the drugs. And then they say ‘oh you know, I don’t feel I can go back to work’, I agree, and I have to say, ‘oh well you will have to come back and see the Doctor’. Or I’ll have to go off and find a Doctor, which is really time-wasting.”* Nurse304

Conversely, the physiotherapist who did not agree with natural role progression claimed an extended role would result in increased workload. Some GPs raised concerns over a ‘just-checking’ ideation, whereby nurses and physiotherapists would create more work for GPs by requesting case discussions prior to certification decisions:

*“…if the way that nurses deal with everything else was to do with it, then I don’t think you would get a – it would just be blank cheques being written, or else they’d always refer them to a doctor…”* GP528

The use of protocols and guidelines to assist certification decisions by non-medical staff was suggested to mitigate increased GP workload. Each professional group raised concerns over the potential for patients to manipulate a new extended role system:

“…when you introduce multiple people doing the same thing there’s always a potential for inter-observer variation. I think the guy’s fine and fine to go to work. The physio doesn’t. And there may be a little bit of playing one side off [against] the other. The guy doesn’t want to go back to work and: ‘Well, ***** you. If you're not going to give me a line I'm going to go to the physio.” GP757

Respondents linked manipulation with increasing demand for physiotherapist and nursing services and possible inappropriate certification practice.

### Control and responsibility

Each group used the perceived professional hierarchy to express their professional legitimacy claims differently. The ability to control the form and content of clinical work is a central tenet of the medical profession generally and a significant guiding principal for GPs. GP narratives reflected this through using words and phrases including *“oversee”, “like to know what is going on”* and *“being the central coordinator”.* Some GPs refrained from talking about control, preferring instead to use complicit status claims through phrases including *“theoretically”* and *“some people”*.

Nurses used the professional hierarchy and existing GP authority to protect their responsibility for patient care and therefore justify role extension, referring to GPs as *“back up*”. Physiotherapists saw role extension as an opportunity to strengthen their professional status as autonomous respected practitioners using words including *“empower”* and *“advantage”.* All GPs highlighted conflicts of duty to the patient and to the State during work-and-health related consultations and the impact on the doctor-patient relationship. GPs were aware of the possible perception of “*dumping”* their least desired tasks. One physiotherapist was particularly wary of this delegation for little reward. Both GPs and nurses referred to the necessity of protocols and guidelines to inform nurse-led certification practice, although each profession's views had different underpinning reasons. GPs advocated guideline use to ensure ‘appropriate’ certification and to exercise control over their nursing colleagues’ practice:

*“…they’re [practice nurses] very good actually because they don’t go anywhere beyond their competence, which you would expect anyway…they’re entirely willing to take on the policies and the principles that we’ve set them.”* GP528

The less experienced nurses appeared more risk-averse, reluctant to take on added responsibility and subsequent accountability. Therefore, protocols were referred to as a means of deferring responsibility to the GP and offering *“protection”.* Physiotherapists did not refer to the use of protocols or guidelines during their interviews and GPs did not relate the use of guidelines to physiotherapists’ extended role, perhaps indicating their superior status in the eyes of GPs.

## Discussion

Extending the authority to certify sickness absence beyond the medical profession in primary care is not a simple matter of addressing organisational obstacles. Role extension is underpinned by the sociological theories of professional identity and boundary work. Respondents employed legitimacy claims to support their views on extension of the sickness certification role beyond the medical profession. Respondents generally supported the concept of role extension for sickness certification, although this support came with conditions including recognition (physiotherapists), use of guidelines (nurses) and maintenance of overall patient care control (GP). Rejections of the extended role concept were based upon the perceived challenges to GPs’ dominance, the apparent inability of non-medical professionals to practice holistically and their lack of access to full medical records, the potential for system manipulation and increased GP workload.

The sociological literature on professional boundaries has been dominated by analyses of the way professions use ‘science’ to differentiate their unique contribution to patient care as superior to the contribution of other groups or professionals. As noted previously, clinicians may adopt a discourse of ‘science’ as means of strengthening their own credibility in the eyes of their colleagues or as a means of presenting their own skills as superior [14]. Our professionals identified four overlapping legitimacy claims which were used in a variety of ways to support or reject role extension; condition specific legitimacy, holistic care, system efficiency, and, control and responsibility. These discourses were used interchangeably by each of our professions giving rise to a mixed picture which revealed diverse opinions about the appropriateness of service redesign in relation to sickness certification practice.

GPs sought to maintain control over the sickness certification process with claims to be best placed to manage ‘complex’ health problems in patients with knowledge of their specific healthcare needs, and by using a more holistic approach than the other professional groups. GPs advocated role extension to nurses only if conducted under supervision and with the aid of clear protocols, or to save GP time by streamlining the more ‘simple cases’ which did not require a GP’s expertise. Physiotherapists however voiced support for role extension, claiming to possess specialist knowledge of musculoskeletal problems, a common cause for work absence. Although they did not claim to practice holistically, they perceived role extension would improve system efficiency as patients would not need to visit a GP as frequently, helping to reduce their workload. In addition, physiotherapists could spend more time with patients to address work absence difficulties and the role extension concept offered physiotherapists the opportunity to extend their skills and perhaps professional status (*see* Sanders et al.) [52]. Nurses also claimed to have more time to spend with patients and perceived role extension as a natural role progression. They claimed to offer greater accessibility to healthcare than could be offered by GPs, though some would only discharge such a role in the presence of clear protocols to guide their decisions. GPs’ overall control over patient records deprived nurses and physiotherapists of information about patients’ background and medical history; a critical requirement for making sickness certification decisions.

To summarise the findings, two overarching trends are evident in the data. First, the claim to specialist skill or knowledge by GPs and physiotherapists to deal with sickness certification decisions was a key factor in determining role extension; this was most prevalent in the claims made by GPs and physiotherapists, with the former largely resisting role extension, and the latter supporting it. The second trend related to widespread use of organisational and system efficiency discourses. All three professions utilised the system efficiency discourse, though most strikingly it was used by nurses and physiotherapists, each claiming that role extension would enhance system efficiency and therefore patient care. In relation to the field of heart failure, Sanders and Harrison [53] also found that occupations lower down the ‘status hierarchy’ in a hospital setting (eg. specialist nurses) predominantly used a system efficiency claim to differentiate their specific contribution to patient care from cardiologists and geriatricians, who did not have time to address patients’ information needs and provide preventative care.

Neither physiotherapists nor nurses explicitly used the discourse of ‘medicine’ or ‘science’ to differentiate themselves from GPs, as in Foley and Fairclough’s [21] study which reported that midwives used a discourse of ‘medicine’ to attempt to establish themselves as equal to doctors, because they too used ‘science’ in their work. Both occupations referred to the *practical* benefits they could bring to primary care through enhancing system efficiency and thus their global contribution to patient care. The close proximity of nurses to GPs in clinical practice may also have had an influence on their claims, with nurses utilising only a managerial discourse of system efficiency without claiming to possess the same technical expertise as GPs. Perhaps seeking only to show how they could add value to an existing set of practices rather than replacing or upstaging the current GPs’ role. They claimed to have more time to dedicate to patients, but stopped short of espousing superior knowledge claims. Physiotherapists however used their technical expertise in the management of musculoskeletal problems to distinguish their contribution from GPs as potentially superior. They adopted both the language of technical skill and system efficiency to support role extension. On the whole physiotherapists and nurses utilised a largely ‘proactive’ stance emphasising their positive contribution to sickness certification. GP’s claims were largely ‘defensive’; attempting to exclude nurses and physiotherapists from the jurisdiction of sickness certification practice, whilst allowing minor adjustments to the current system such as through ‘delegation of dirty work’, a strategy used to reinforce the medical model of dominance by doctors through determination of nursing and physiotherapy boundaries [54].

The equation of a holistic approach with access to GP held information is not widely reported in the literature. It could be argued that physiotherapists should not be precluded from delivering holistic care, including sickness certification, on the basis of information access since the amount of information known is a matter between healthcare professionals and patients.

## Conclusions

The use of protocols to guide tasks appropriate for nurse-delegation in primary care is known [7]; the present study suggests that GPs also used protocols and guidelines to exert control through defining occupational boundaries. Extending the authority to certify sickness absence to nurses and physiotherapists in primary care is a complex undertaking. Although the majority of respondents supported the concept, the views of the ‘sceptics’ are equally important. For instance, implementing such a change is not simply a case of overcoming practical and organisational barriers. Our study demonstrates that in relation to role extension, professions hold deeply-entrenched values that are underpinned by professional identities. Further exploration of these values is required to understand specific professional responses to organisational change and aid planning and implementation of future primary care role extension, including the task of sickness certification.

### Study limitations

Respondents with a range of characteristics were interviewed to ensure a spread of opinions was captured. The relatively small sample risks overlooking alternative views, particularly in the physiotherapist group where some additional insights continued to emerge during the final interview. However, these were not related to the ‘core’ themes of legitimacy claims and boundary work explored in the current analysis. Thus we are reasonably confident that the main subject of analysis presented in this paper has been explored in depth without key issues having been missed or overlooked. Physiotherapist recruitment through associations with the host Research Centre raises the possibility that responses were tailored to avoid impact upon future relationships. However, this is unlikely given the range of physiotherapist views elicited. Most physiotherapists had received specialist musculoskeletal training, a sampling strength since the second most common reason for sickness certification is musculoskeletal ill-health [55].

The interviewer’s occupation, a GP-trainee, was disclosed prior to interview commencement. This may have influenced interviews, for example one physiotherapist used detailed clinical language when referring to musculoskeletal pain and the impact on sickness certification, perhaps to present themselves as technically competent to the GP trainee researcher. In addition a nurse interviewee expressed some ‘negative’ views towards GPs. Telephone interviews may restrict rapport development and recognition of non-verbal cues. In this study, the degree of anonymity afforded through telephone use noticeably encouraged participation in a potentially sensitive topic area [56]. We are also aware that the relatively low response rate from GPs may lead to the exploration of views from a select group of respondents, although the recruitment of GPs has always presented this dilemma in other qualitative (and quantitative) research. We do not seek to claim that our findings are ‘representative’ or generalizable to the entire population of GPs, nurses and physiotherapists working in the UK, but that the findings identify important insights some (if not most) are likely to be held by a wider group of clinicians. Further in-depth qualitative research on this topic is therefore required to build on the themes presented here.

## Competing interests

There are no competing interests to declare.

## Authors’ contributions

VW carried out the qualitative fieldwork, took a lead in the analysis and drafted the manuscript. TS contributed to the analysis, conception of the manuscript and writing up. JR contributed to the analysis, conception of the manuscript and writing up. GWJ contributed to the analysis and writing up. CJ contributed to the analysis and helped to draft the manuscript. CM contributed to the analysis, conception of the manuscript and writing up. All authors read and approved the final manuscript.

## Pre-publication history

The pre-publication history for this paper can be accessed here:

http://www.biomedcentral.com/1471-2296/15/100/prepub
